# Variation of Saturation Across Hue Affects Unique and Typical Hue Choices

**DOI:** 10.1177/2041669519872226

**Published:** 2019-09-12

**Authors:** Christoph Witzel

**Affiliations:** Justus-Liebig-Universität, Gießen, Germany

**Keywords:** chroma and saturation, colour naming, colour categories, linguistic relativity

## Abstract

Most studies on colour categorisation and many studies on unique hues have used samples of maximally saturated Munsell chips that vary in saturation across hue. Here we show that observers’ choices of category prototypes and unique hues depend on the variation of Munsell chroma across hue. Both unique hue and prototype choices were shifted towards the more saturated hues in the respective stimulus set. This effect of saturation may explain cross-cultural regularities in colour categorisation. More generally, these findings highlight the importance of controlling saturation when measuring colour categories and unique hues.

## Introduction

Studies on colour categorisation have focused on the categorisation of hue and lightness and have neglected the role of saturation and chroma (for review, see Witzel, 2018a, 2018b). Most studies on colour categorisation have been using maximally saturated Munsell chips, which strongly vary in their saturation across hue and lightness. Similarly, many studies on colour appearance have used maximally saturated Munsell chips to determine unique hues (e.g., Kuehni, 2014; Kuehni, Shamey, Mathews, & Keene, 2010; Logvinenko & Geithner, 2015).

Maximally saturated Munsell chips have local maxima of saturation around the prototypes of English colours terms (Witzel, 2018b; Witzel, Cinotti, & O’Regan, 2015). Colour categories and category prototypes are defined along all three dimensions of colour perception, including saturation (e.g., Figure 8 in Olkkonen, Witzel, Hansen, & Gegenfurtner, 2010). Observers tend to choose saturated rather than desaturated colours as prototypes of chromatic colour categories (see also Bonnardel et al., 2016). The tendency to choose saturated colours as prototypes could explain why observers from fundamentally different languages choose Munsell chips as prototypes that coincide with English prototypes (Witzel, 2018b). This idea is supported by the observation that the variation of saturation in the classical set of maximally saturated Munsell chips is correlated with prototype choices and with colour naming consistency within and across languages (Lindsey, Brown, Brainard, & Apicella, 2016; Witzel, 2016, 2018b; Witzel et al., 2015).

However, the correlations do not establish a causal relationship between saturation and categorisation and could instead be pure coincidence. To test for a causal relationship, we investigated whether variation of saturation across hue influences which hues are chosen as category prototypes. We created two stimulus sets that only differed in how saturation varied across hue; we measured category prototypes, unique hues, and binary hues with each of the two stimulus sets; and we tested whether these measurements differed across stimulus sets.

We predicted a trade-off between hue and saturation in prototype choices: When the typical hue is not available at highest saturation, observers compromise their hue selection in order to select a colour with high saturation. If this is true, observers should choose colours of different hue as prototypes depending on the stimulus set. In contrast to category prototypes, unique hues are exclusively defined by hue, not by saturation. Based on this definition, we predicted that unique and binary hue choices should not be affected by varying saturation across hues.

## Method

### Participants

Six men and 17 women (*M* = 24.6 ± 6.6 years) took part in the experiment. Participants were employees and students at the Justus-Liebig-University in Gießen. No participant was red–green colour deficient as tested with the Ishihara plates. Observers were German speakers, and the experiment was conducted in German (instructions and colour terms reported here are translations from German).

### Apparatus

Observers sat at a table in front of a window; their face was directed towards the window. Stimuli were illuminated by the natural daylight coming from the window. The lighting conditions were similar to the ones described in detail in a previous study (Olkkonen et al., 2010). The table was covered with a grey fabric to control local contrast. The examiner sat to the right of the participant and presented the stimuli in each trial on the grey fabric.

### Stimuli

Note that we held lightness constant in each stimulus set, implying that the variation of chroma was equivalent to a variation of saturation in our stimulus sets (cf. Chapter 4 in Fairchild, 2005). We defined our stimuli as Munsell chips because our experiment was aimed at explaining the correlations found between Munsell chroma and colour categorisation (Lindsey et al., 2016; Witzel, 2016, 2018b; Witzel et al., 2015). Munsell colour chips were taken from the Glossy Collection (Munsell Color Services, 2007). We prepared two sets of stimuli that differed systematically by the variation of Munsell chroma across hues. Lightness and hues were the same in both sets and were determined based on previous studies.

The previous studies provided preliminary typical, unique, and binary hues that were used as *seeds* of our stimulus sampling. Hence, the seeds represent predictions under the null hypothesis that saturation does not affect prototype, unique, and binary hue selection. We determined hue transitions around those seeds to measure typical, unique, and binary hues. Munsell Values were identical for every chip in the two samples of a seed. For each category, the Munsell Value of the chips was the typical lightness of the category prototype previously measured (see Figure 8 in Olkkonen et al., 2010).

**Figure 1.**
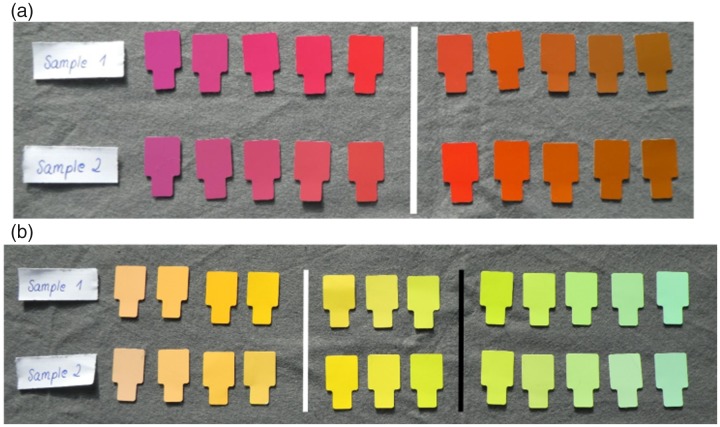
Samples for measuring typical and unique red (a) and yellow (b). In each sample (row), hue varies at constant lightness from left to right. The vertical white and black lines within each sample indicate the location of typical red (a) and yellow (b) and the boundary between yellow and green (b) according to Olkkonen et al. (2010). In panel (a), the upper row shows the “blue-biased” Sample 1. In that sample, saturation (i.e., Munsell chroma) is higher for the five Munsell chips to the left bluish than for the five chips towards the right yellowish hue direction. Sample 2 in the lower row is yellow-biased with higher saturation to the right yellowish hue direction than to the left. In panel (b), Sample 1 is red-biased and Sample 2 green-biased for the measurement of typical and unique yellow. They are green-biased (Sample 1) and yellow-biased (Sample 2) for the measurement of binary yellow–green because the saturation–hue relationship reverses at the binary hue (black line).

Figure 1 illustrates the variation of Munsell chroma and hue across the two stimulus sets with the examples of red and yellow. Table A1 provides details for all stimulus sets. In Set 1, hues counterclockwise (decreasing Munsell hue) to the seeds were determined to have higher Munsell chroma than those clockwise (increasing Munsell hue) to the seeds. For example, Munsell chroma was higher for bluish than for yellowish hues around typical red in Set 1 (blue-biased) and higher for yellowish than for bluish hues (yellow-biased) in Set 2 (cf. Figure 1(a)). The differences in saturation across stimulus sets were four steps of Munsell chroma for red, orange, yellow, and green, and two steps of Munsell chroma for all other stimulus sets (see Table A1 for details). As roughly illustrated by comparing the samples in Figure 1, these differences are rather small and not obvious.

Apart from the change at the seed, we tried to keep Munsell chroma constant within a stimulus set. At the same time, we also wanted colours that are sufficiently saturated to unambiguously belong to the chromatic categories rather than to grey. It is impossible to obtain Munsell chips that have at the same time high and completely constant Munsell chroma because of the strong variation of maximal available Munsell chroma across Munsell hue (Witzel et al., 2015). As a compromise between the two criteria, we allowed for Munsell chroma to slightly vary within a stimulus set. We found this acceptable because the variation of chroma was comparable to the one in the set of maximally saturated Munsell chips used in classical colour naming and unique hue studies.

There were separate hue transitions for each seed, for example, a separate range of 10 hues for measuring unique and typical red (see Figure 1(a)) and a separate range of 10 hues for typical orange and unique red–yellow. There were three exceptions to this. The yellow hue range was combined with the binary yellow–green range as illustrated by Figure 1(b). The blue hue range was combined with the purple hue range for measuring typical purple and binary blue–red, and the green range was combined with the green–blue range. Hence, there were overall 14 different stimulus sets, namely seven different hue ranges with two stimulus sets each. There was a hue range for (1) pink; (2) red; (3) orange and binary red–yellow; (4) yellow and binary yellow–green; (5) green and binary green–blue; (6) blue, purple, and binary blue–red; and one for brown (7).

**Figure 2.**
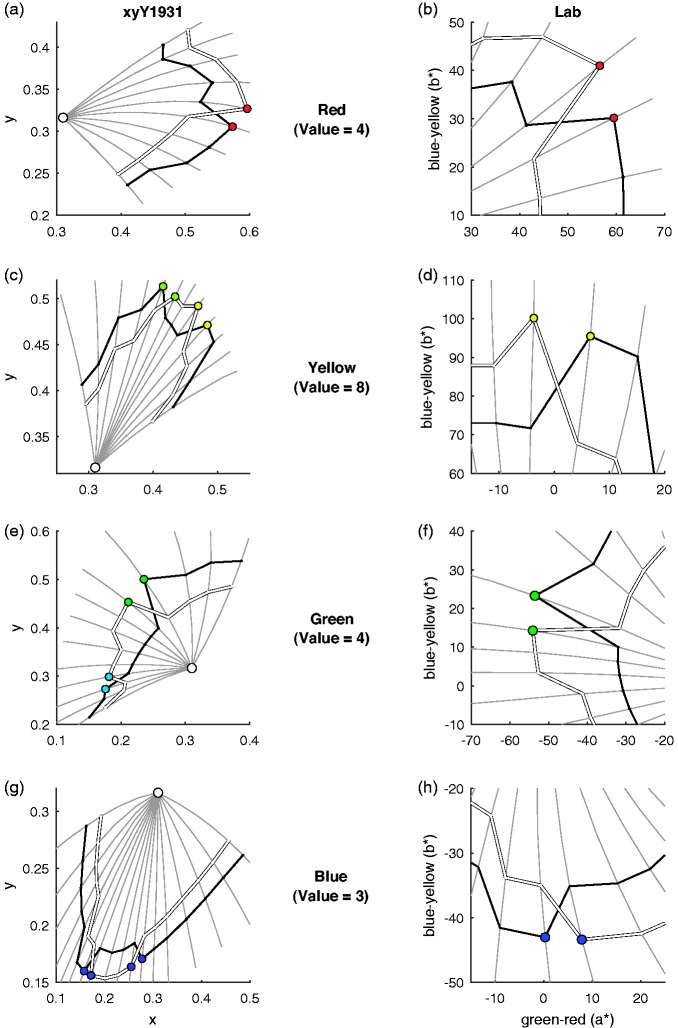
Interactions between hue and saturation. Black and white curves show the chromaticity coordinates (first column) and CIELAB coordinates (second column) for the red (a–b), yellow–green (c–d), green–blue (e–f), and blue–purple (g–h) stimulus sets (cf. Table A1). Chromaticity coordinates for the Munsell chips correspond to standard illuminant C (white disc). Grey curves show different levels of Munsell chroma for constant Munsell hues and illustrate the Abney effect. Coloured discs indicate which colour observers would choose as the prototype and unique hue if their choices were affected by saturation. For prototype and unique hue choices, these predictions clearly held.

The seeds for category prototypes and unique hues corresponded to the mode prototype choices in the naming study (largest disks in Figure 8 of Olkkonen et al., 2010). The hue of the seeds for red, yellow, green, blue, orange, and purple also corresponded to the hues reported for unique hues and binary red-yellow and blue-red (Table 3 in Kuehni, 2014; Table 1 in Kuehni et al., 2010). The seeds for binary yellow-green and green-blue were taken from Table 1 in Kuehni et al. (2010). According to previous measurements, choices of unique red may be slightly more bluish than typical red choices (Witzel & Franklin, 2014; Witzel, Maule, & Franklin, 2019). For this reason, we prepared two different seeds for red. Thirteen participants were presented a hue range with a seed between Munsell 5R and 7.5R, which is the most frequent (mode) choice for typical red (Olkkonen et al., 2010); the other 10 observers were shown a hue range with a seed between Munsell 2.5R and 5R, which was closer to more bluish choices of unique red (Kuehni, 2014). Figure 2 illustrates the resulting sets for red, yellow, green, and blue in the 1931 chromaticity diagram and in CIELAB space.

### Procedure

In one trial, the chips of one of the 14 hue ranges were presented in an unordered (random) arrangement to the observer. The observer was asked to either choose one of the eight chromatic category prototypes, one of the four unique hues, or one of the four binary hues, depending on the experimental condition.

The experiment had four blocks. Two blocks measured unique and binary hues with stimulus Set 1 and Set 2, respectively; the third and fourth blocks measured category prototypes for stimulus Set 1 and Set 2, respectively. The instructions for unique hue selection were for the example of unique red: “Which red is neither yellowish nor bluish? Which red is the pure red?” Those for the binary hue selection were as follows: “Which of these colours is as reddish as yellowish? Which of these colours contains 50% red and 50% yellow?” The following were the instructions for choosing the category prototype: “Which red is the typical red, the best example for the colour category red? Which red is redder than any other colour?”

The two blocks for unique and binary hues were always completed before the two blocks for category prototypes in order to minimise the interference of the category prototype selection on the unique and binary hue selection. In each of the first two blocks, the trials for unique hues were completed before those for binary hues. Apart from that, the assignment of the stimulus sets to each block and the trials within each block were randomised. There were no repeated measurements for the same condition and stimulus. The experiment took 20 to 30 minutes in total.

## Results and Discussion

Figure 2 (coloured circles) illustrates the predictions with the examples of red, yellow, green, and blue: If saturation affects prototype, unique, and binary hue choices, observers are expected to select colours with comparatively high saturation, implying that the hues of the colours differ between the two stimulus sets. Table A2 provides average choices for each condition, and individual data can be accessed through Zenodo (https://doi.org/10.5281/zenodo.3357670).

**Figure 3.**
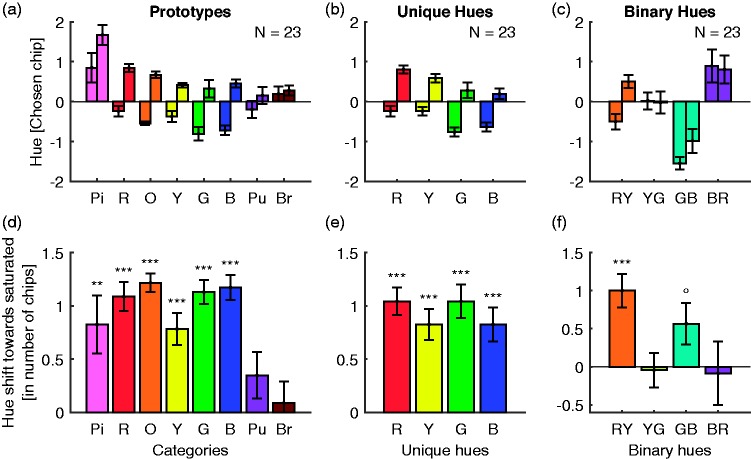
Results. The first row shows the choices of category prototypes (a), unique hues (b), and binary hues (c). The *x*-axes indicate the hue ranges and stimulus sets. The *y*-axis represents hue measured as the number of the Munsell chips away from the seed of the respective hue range. A unit corresponds to a Munsell hue step of 2.5. A value of zero indicates the seed of the hue range. A positive (negative) value corresponds to a clockwise (counterclockwise) hue shift away from the seed. The two bars for each hue range correspond to the first and second stimulus set. The lower row (d–f) illustrates the differences between the selection in the first and second stimulus set, that is, the differences between the corresponding two bars in the first rows. Positive differences represent shifts towards more saturated colours. Error bars indicate standard errors of mean; symbols above the bars report *p* values for differences from zero in paired *t* tests. ***<.001. **<.01. *<.05. °<.1.

Figure 3 illustrates the main results. To account for the different sizes of the hue ranges (cf. Figure 1(a) vs. (b)), we express the hue chosen by observers as the difference from the seed of the hue range. For better illustration, we swapped Set 1 and Set 2 for purple/blue–red, yellow–green, and green–blue because their variation of Munsell chroma across hue was reversed (see Method section). In this way, all bars in Figure 2 have the same meaning for our tests and hypotheses. We report effects sizes as Cohen’s *d* for *t* tests and partial eta squared for repeated measurements analyses of variance (RMAOV).

### Category Prototypes

Figure 3(a) illustrates prototype choices for each stimulus set relative to the preliminary prototypes that were taken as seeds of the hue ranges. Figure 3(d) visualises the differences between stimulus Set 1 and Set 2.

To test whether colour choices differed between the first and the second stimulus set, we calculated a two-way RMAOV with the factors hue type (pink, red, . . . brown) and stimulus set. There was a main effect of stimulus set—*F*(1,22) = 297.7, *p* < .001, *η*^2^ = 0.93—indicating the effect of saturation on prototype choices. The main effect for hue type—*F*(7,154) = 8.8, *p* < .001, *η*^2^ = 0.29—implied that prototype choices (averaged across stimulus sets) deviated from the preliminary prototypes (i.e., the seeds). There was also a significant interaction—*F*(7,154) = 5.3, *p* < .001, *η*^2^ = 0.70—indicating that the difference between stimulus sets was stronger for some (e.g., red, orange) than for other prototypes (brown, purple).

As can be seen from the positive bars in Figure 3(d), prototype selections were all on average shifted towards higher saturation. Post hoc paired *t* tests confirmed that prototype choices differed significantly between the first and the second stimulus set for pink, red, orange, yellow, green, and blue (all *p* < .007, min. Cohen’s *d  = * 0.6). These results were still significant after a Holm–Bonferroni correction for eight tests. Average choices for purple and brown prototypes showed the same tendencies as the other prototype choices; however, the difference between the two stimulus sets was not significant—*t*(22) = 1.6, *p* = .13, *d* = 0.3; *t*(22) = 0.4, *p* = .68, *d* = 0.1.

We also explored the main effect for hue type through post hoc *t* tests. They showed that pink and red prototype choices were systematically more yellowish than the seeds—min. *t*(22)  = 3.3, both *p* < .003, min. *d* = 0.7. This observation fully agrees with German prototype choices in the previous study (Figure 8 in Olkkonen et al., 2010), according to which the modes of prototype choices for pink and red (5RP and 7.5R) are on the yellowish instead of the bluish side of the seed (cf. Tables A1 and A2).

We examined whether there was a difference in hue choices between the two groups of observers who selected red from slightly different hue types. Both groups yielded a significant shift towards more saturated colours in the comparison between stimulus Set 1 and Set 2—*t*(9)  = 5.3, *p* < .001, *d* = 1.7; *t*(12) = 8.1, *p* < .001, *d* = 2.3. A *t* test comparing the two groups showed that this effect was stronger in the group with the slightly more bluish seed—*t*(21) = 2.1, *p* = .046, *d* = 0.85. These observations show that small variations of the seed of the stimulus set modulates the main effect of saturation on hue but does not completely counteract this effect.

Taken together, these results clearly show that for six out of eight categories observers choose the more saturated colours as prototypes in both stimulus sets, implying that the chosen Munsell chips differed in hue. The only exceptions were purple and brown. The lack of an effect for brown is understandable if we consider the unimportance of saturation for the brown category. Brown colours have generally low levels of saturation compared to other chromatic categories, indicating that high saturation is not a characteristic feature of brown. For this reason, typicality judgements for brown might be less affected by saturation. It is not clear whether a similar argument applies to purple because purple contains many highly saturated colours (e.g., Figure 8 in Olkkonen et al., 2010).

**Figure A3.**
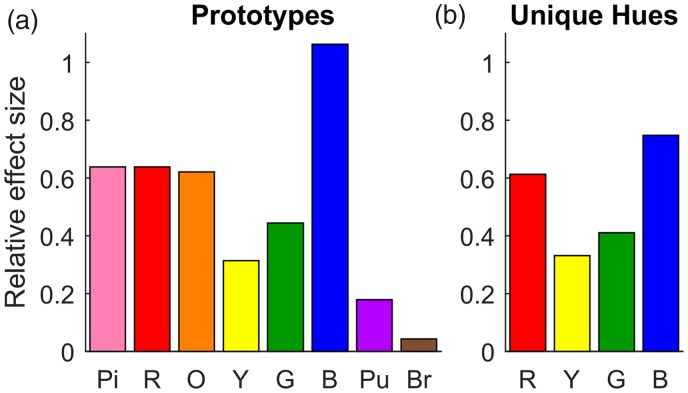
Relative effect sizes. The *y*-axis represents the relative effect size, that is, the hue shift divided by the difference in saturation. For this, we represented colours in CIELAB (Figure 2). For each hue type, we determined the Euclidean distance between the two colour chips selected for each of the two stimulus sets (coloured dots in Figure 2). Since this distance represents a discrete difference between two chips we multiplied that distance with the average number of chips across observers (Figure 3(d) to (e)). We then determined the distance between the hues at the two levels of saturation occurring across the two stimulus sets (distance of the coloured discs to the less saturated stimuli on the same grey hue line in Figure 2), and averaged the two distances. The relative effect size is then the hue difference divided by the average difference in saturation. A value of 1 indicates that the colour difference resulting from the effect on hue is as large as the colour difference due to the manipulation of saturation across stimulus sets. Apart from that, format is as in Figure 3: (a) category prototypes and (b) unique hues. Note that many of the observed colour shifts across stimulus sets involve at least 50% (pink, red, orange, and blue) of the experimental manipulation of saturation that caused the effects.

The hue shifts of the other six categories were on average about 1 chip (2.5 Munsell hue steps), which is a clearly visible (suprathreshold) colour difference (Figure 3(d)). This hue shift resulted from an experimental manipulation of saturation across the two stimulus sets of only 2 to 4 levels of Munsell chroma (cf. Method section). As a result, the hue shift relative to the difference of saturation between the stimulus sets is quite high (e.g., over 100% for blue; cf. Figure A3 for illustration). The variation of saturation in the classical set of maximally saturated Munsell chips is much higher than in our stimulus sets. Hence, still larger effects can be expected in typical studies on colour naming and unique hues that use those classical stimuli.

The strong evidence for an effect of saturation on category prototype choices shows that the correlations between prototype choices and Munsell chroma observed previously (Witzel et al., 2015; Witzel, 2016; Witzel, 2018b) were not a coincidence but reflect an effect of saturation on typicality judgements. The effect of saturation may explain cross-cultural regularities in colour categorisation (Regier, Kay, & Cook, 2005) as well as the perceptual salience of typical red, yellow, green, and blue (Regier, Kay, & Khetarpal, 2007).

Previously we found that high saturation around the typical colours of English colour terms is a peculiarity of the set of maximally saturated Munsell chips, not a property of human colour perception (Witzel & Franklin, 2014; Witzel et al., 2019). The effect of saturation on prototype choices suggests that the perceptual salience of English category prototypes might at least partly depend on the stimulus sample. More generally, the present results highlight that saturation is an important dimension of colour categorisation. In as far as typicality is related to the strength of category membership, these results may also elucidate the correlations between saturation and category consistency (Lindsey et al., 2016; Witzel, 2016, 2018b).

### Unique Hues

The centre column of Figure 3 illustrates unique hue choices (Figure 3(b)) and the differences between choices for stimulus Set 1 and Set 2 (Figure 3(e)). As for prototypes, the positive bars in Figure 3(e) indicate that unique hue choices were shifted towards the more saturated hues. The difference between Set 1 and Set 2 yielded a significant main effect in the two-way RMAOV—*F*(1,22) = 194.2, *p* < .001, *η*^2^ = 0.90. There was also a main effect of hue type—*F*(3,66) = 6.9, *p* < .001, *η*^2^ = 0.24, but there was no significant interaction—*F*(3,66) = 0.7, *p* = .59, *η*^2^ = 0.03.

Post hoc *t* tests confirmed that the hues of the chosen colours were shifted towards the more saturated colours for unique red, yellow, green, and blue—all *t*(22)>5.1, all *p* < .001, all *d* > 1.1. These results were still significant after a Holm–Bonferroni correction for four tests.

Post hoc *t* tests for the main effect of hue type showed that choices for unique red were more yellowish—*t*(22) = 3.0, *p* = .006, *d* = 0.6—and those for unique yellow—*t*(22) = 2.2, *p* = .04, *d* = 0.4—and blue—*t*(22) = –2.3, *p* = .03, *d* = –0.5—more greenish than the preliminary unique hues taken as the seed (cf. Tables A1 and A2). As for the corresponding effects for prototypes, these observations are in line with unique and prototype hues obtained in previous studies (Kuehni, 2014; Olkkonen et al., 2010).

The two groups with the slightly different red stimulus sets both chose more saturated colours as unique red—*t*(9) = 4.1, *p* = .003, *d* = 1.3; *t*(12) = 12.0, *p* < .001, *d* = 3.3. In contrast to prototype choices, the effect of saturation did not differ significantly between the two groups—*t*(21) = 1.0, *p* = .31, *d* = 0.4.

We also compared unique hue choices to choices of typical red, yellow, green, and blue. For this, we averaged the selected hues across the two stimulus sets and tested whether typical and unique hues differed in a paired *t* test. There was no significant difference in any of the four comparisons (all *p* > .11). Instead, prototypes and unique hue choices were correlated across observers for red, yellow, green, and blue in both sets—all *r*(21) > 0.51, all *p* < .02. The only exceptions were yellow—*r*(21) = .36, *p* = .09—and blue in the second set—*r*(21) = .10, *p* = .63—probably due to comparatively low variance of prototype choices across observers. The similarities between prototype and unique hue choices is generally in line with previous measurements, except for red (Witzel & Franklin, 2014; Witzel et al., 2019). The absence of a difference between typical and unique red may be explained by the fact that our stimuli here were much more saturated (maximum or close to maximum) than those of the previous studies (Witzel & Franklin, 2014; Witzel et al., 2019), and low saturation may differentially affect prototypes and unique hues.

In contrast to our initial hypothesis, these results clearly show that unique hue judgements are affected by the variation of saturation across hues. Observers shift the hue of their colour choice so as to obtain more saturated colours.

These effects are different from the known variation of unique hue measurements across constant levels of chroma (Shamey, Zubair, & Cheema, 2019; Witzel & Gegenfurtner, 2018; Xiao, Pointer, Cui, Chauhan, & Wuerger, 2015), and in particular from the *Abney effect* (e.g., Burns, Elsner, Pokorny, & Smith, 1984; Mizokami, Werner, Crognale, & Webster et al., 2006). While the Abney effect consists of a change of perceived hue with varying saturation within a hue, our results show a change of hue choices when varying saturation across hue (cf. Figure 2). The Abney effect approximately corresponds to the bent curve of chromaticity coordinates for Munsell hue at different levels of Munsell chroma (e.g., Newhall, Nickerson, & Judd, 1943; Pridmore, 2007; see also Figure 6.6–6.7 in Fairchild, 2005). So, the Abney effect consists of a change into one particular hue direction with increasing saturation (cf. curves of constant Munsell hue in the left column of Figure 2). In contrast, our results imply an effect of saturation in opposite hue directions in the two stimulus sets. For example, the choice for typical and unique red is more bluish in one stimulus set, and more yellowish in the other, even though the choices in both sets are most saturated (right column of Figure 2). These observations further undermine the idea that unique hues are independent of saturation.

However, it is not clear whether our results can be generalised to other approaches to measure unique hues. In our method, the difference between typicality and uniqueness merely depends on instructions, which involved colour names. These instructions might well lead the observer to consider linguistic colour categories when completing the unique hue selection. It would be interesting to examine whether saturation influences unique hue judgements in other methods, such as hue scaling (e.g., Sternheim & Boynton, 1966) or partial hue matching (e.g., Logvinenko & Geithner, 2015), and with stimulus samples other than Munsell chips.

### Binary Hues

The last column of Figure 3 illustrates the binary hue choices (Figure 3(c)) and their differences between stimulus sets (Figure 3(f)). Average differences vary depending on the binary hue. There was a main effect of stimulus set—*F*(1,22) = 5.3, *p* = .03, *η*^2^ = 0.19, a main effect of hue type—*F*(3,66) = 15.7, *p* < .001, *η*^2^ = 0.42, and a significant interaction—*F*(3,66) = 3.1, *p* = .03, *η*^2^ = 0.13. Post hoc *t* tests revealed a significant shift of red-yellow towards higher saturation—*t*(22) = 4.4, *p* < .001, *d* = 0.92—which is also significant after a Holm–Bonferroni correction for four tests. This result for red–yellow is equivalent to the one found for typical orange. Hue shifts for other binary hues were not significant—highest *t*(22) = 2.0, *p* = .06, *d* = 0.42 for green–blue.

Post hoc *t* tests exploring the main effect of hue type showed that the seeds for binary green–blue and binary blue–red were too bluish. Observers systematically chose more greenish colours as binary green–blue—*t*(22) = –6.3, *p* < .001, *d* = –1.3—and more reddish colours as binary blue-red—*t*(22) = 2.6, *p* = .02, *d* = 0.54. Finally, we calculated *t* tests that compared choices for typical orange with those for binary red–yellow, and choices for typical purple with those for blue–red, but there were no significant differences (both *p* > .07).

In sum, unlike the results for prototypes and unique hues, those for binary hues are inconsistent. It is possible that the effect of saturation on binary hue choices was weakened because the seed of the hue range was too different from the actual binary green–blue and blue–red. Measuring the role of saturation for several intermediate hues, for example, through hue scaling (see earlier) might be a promising approach to further investigate the interaction of hue and saturation across colour space.

## Conclusion

Our findings show that the variation of saturation across hue influences which hues observers pick as category prototypes and unique hues. The effect on category prototypes substantiates the idea that cross-cultural patterns of categorisation could be due to the variation of saturation in the set of maximally saturated Munsell chips that is widely used in colour naming studies. More generally, our findings highlight the importance of controlling saturation in research on colour naming and colour appearance.

## Appendix A

**Table A1.** Detailed Stimulus Specifications.

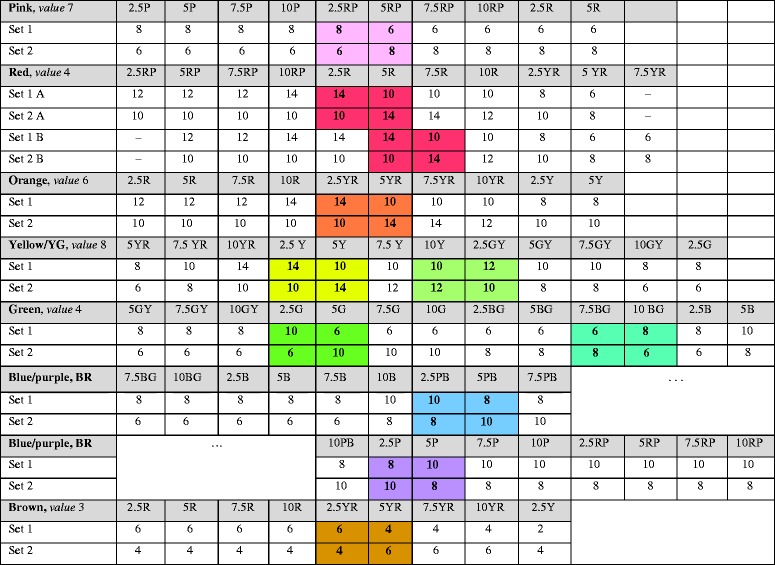

The code in the grey rows indicates Munsell hue. Munsell Value is given in the first column (*value*). The entries in the white cells report Munsell chroma. The two chips on either side of each seed are highlighted by bold type and background colour. Sets A and B for red refer to the different sets used for the two groups of observers (see Method).

### Average Hues

**Table A2.** Average Hues.

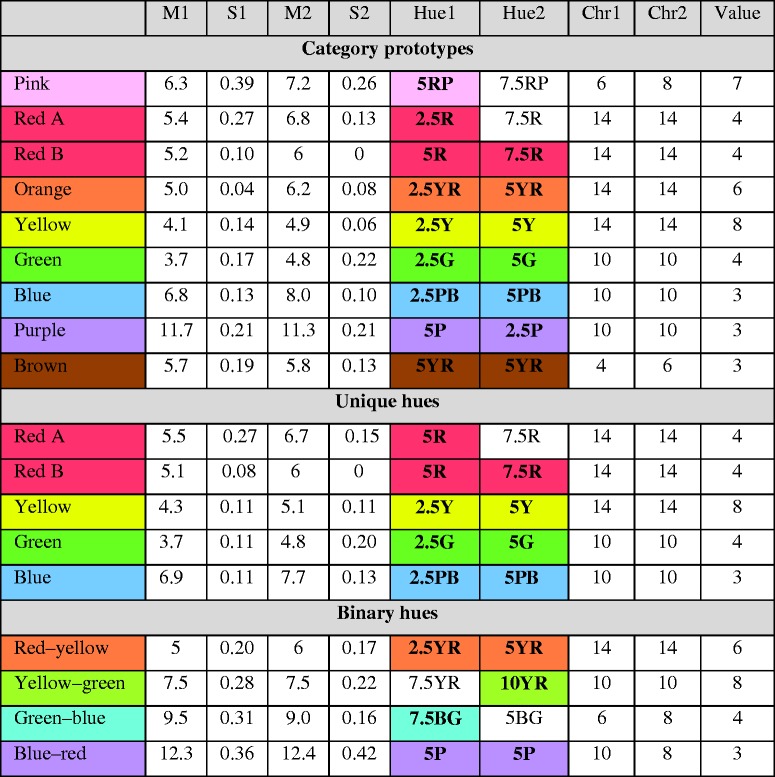

M1, S1, M2, and S2 report the average and standard error of mean of the hue rank in each stimulus set (1 and 2). Hue1, Hue2, Chr1, and Chr2 report the chip that corresponds to the rounded M1 and M2. Value (last column) was constant in each stimulus set. Hues highlighted in bold and colour are adjacent to the seeds of the respective sample.
